# From Bench to Bedside: Translating Cellular Rejuvenation Therapies into Clinical Applications

**DOI:** 10.3390/cells13242052

**Published:** 2024-12-12

**Authors:** Timur Saliev, Prim B. Singh

**Affiliations:** 1 S.D. Asfendiyarov Kazakh National Medical University, Tole Bi Street 94, Almaty 050000, Kazakhstan; 2 School of Medicine, Nazarbayev University, Astana 010000, Kazakhstan; prim.singh@nu.edu.kz

**Keywords:** rejuvenation, senolytics, CRISPR, induced pluripotent stem cells, mesenchymal stem cells

## Abstract

Cellular rejuvenation therapies represent a transformative frontier in addressing age-related decline and extending human health span. By targeting fundamental hallmarks of aging—such as genomic instability, epigenetic alterations, mitochondrial dysfunction, and cellular senescence—these therapies aim to restore youthful functionality to cells and tissues, offering new hope for treating degenerative diseases. Recent advancements have showcased a range of strategies, including epigenetic reprogramming, senolytic interventions, mitochondrial restoration, stem cell-based approaches, and gene-editing technologies like CRISPR. Each modality has demonstrated substantial potential in preclinical models and is now being cautiously explored in early-stage clinical trials. However, translating these therapies from the laboratory to clinical practice presents unique challenges: safety concerns, delivery precision, complex regulatory requirements, ethical considerations, and high costs impede widespread adoption. This review examines the current landscape of cellular rejuvenation, highlighting key advancements, potential risks, and the strategies needed to overcome these hurdles.

## 1. Introduction

Aging is marked by a progressive decline in cellular, tissue, and organ function, driven by a range of molecular and cellular changes collectively known as the hallmarks of aging. These hallmarks include genomic instability, telomere attrition, epigenetic alterations, loss of proteostasis, mitochondrial dysfunction, cellular senescence, and stem cell exhaustion [1,2]. One of the primary hallmarks, genomic instability, results from the accumulation of DNA damage over time. As we age, the body’s DNA repair mechanisms lose effectiveness, allowing genetic mutations to accumulate. These mutations impair cellular function and heighten the risk of diseases such as cancer.

Telomere attrition, or the gradual shortening of the protective caps at chromosome ends, is another contributor to aging [3]. Each time a cell divides, telomeres shorten slightly, eventually reaching a critical length that triggers cellular senescence or apoptosis (programmed cell death). This telomere shortening weakens tissue integrity and plays a role in age-related conditions, such as cardiovascular disease.

Epigenetic alterations are closely linked to these changes, which involve shifts in DNA methylation and histone modifications that alter gene expression patterns [4,5]. Over time, these changes disrupt cellular function, causing loss of identity and contributing to neurodegenerative diseases like Alzheimer’s and Parkinson’s [6].

Aging cells lose proteostasis, becoming less effective at folding and degrading proteins, which leads to dysfunctional protein buildup [7]. This imbalance creates cellular stress and is strongly associated with neurodegenerative conditions, further impairing cellular functionality.

Mitochondrial dysfunction is another hallmark that plays a central role in aging [8]. Mitochondria, the powerhouses of cells, generate energy. However, with age, mitochondrial efficiency declines, leading to reduced energy production and increased oxidative stress. This mitochondrial decline especially impacts high-energy-demand tissues and contributes to metabolic disorders and age-related organ decline [9].

Cellular rejuvenation therapy aims to reverse or mitigate these aging hallmarks, thereby restoring function and extending cellular health span [10,11]. Epigenetic changes, such as chemical modifications to DNA and histones that influence gene expression, accumulate with age and contribute significantly to the aging process [12]. These changes include DNA methylation, histone modifications, and alterations in chromatin structure, leading to a disrupted gene expression profile that promotes cellular aging [13,14].

Epigenetic reprogramming aims to reverse these age-associated changes without causing the cells to lose their differentiated state [15,16]. Partial reprogramming uses Yamanaka factors (Oct4, Sox2, Klf4, and c-Myc) to reset epigenetic changes without inducing a pluripotent state [17,18]. Partial reprogramming has been shown to restore youthful gene expression profiles, enhance DNA repair, and improve cellular function in aging cells [19]. This approach holds promise for age-related diseases by potentially reactivating silenced genes critical to cellular health while repressing harmful age-associated genes [20,21].

As cells age, some enter a state known as cellular senescence: they stop dividing and adopt a pro-inflammatory secretory phenotype, known as the senescence-associated secretory phenotype (SASP), which can lead to chronic inflammation, tissue dysfunction, and degeneration [22,23]. Senescent cells accumulate in aged tissues and are implicated in many age-related diseases, from osteoarthritis to cardiovascular disease [24,25]. Senolytic therapies are designed to selectively target and eliminate these senescent cells, thereby reducing SASP-associated inflammation and improving tissue function. Drugs such as and quercetin, as well as more recent compounds like fisetin, have shown promise in selectively clearing senescent cells, thus reducing tissue inflammation and restoring function [26,27].

Senomorphic therapies, on the other hand, do not eliminate senescent cells but instead modulate their secretory phenotype, reducing the inflammatory impact of SASP while allowing senescent cells to persist [28,29]. These therapies focus on suppressing pro-inflammatory cytokines, growth factors, and proteases within SASP, thereby mitigating their deleterious effects on surrounding tissues. For example, inhibitors of key SASP regulators, such as NF-κB or mTOR, have shown potential in reducing the inflammatory burden without disrupting the beneficial roles senescent cells may play in processes like wound healing and tumor suppression.

Both senolytic and senomorphic therapies aim to alleviate the burden of senescent cells, improving the regenerative potential of aging tissues [30,31]. Additionally, long-term safety and efficacy are critical in rejuvenation therapies. For example, while therapies that eliminate senescent cells (senolytics) have shown promise in clearing harmful cells, there is concern about potential off-target effects and how the body’s immune system might respond over time [32,33]. Emerging strategies for addressing these concerns include developing more selective senolytics that target specific senescent cell markers and employing localized delivery systems to minimize systemic exposure. Additionally, combining senotherapeutics with immunomodulatory agents is being explored to enhance the clearance of senescent cells while minimizing immune-related side effects. The integration of biomarkers for identifying and monitoring senescent cell burden is also critical for advancing these therapies into routine clinical use.

Similarly, mitochondrial-targeted therapies aim to restore cellular energy and reduce oxidative damage, but unforeseen impacts on mitochondrial dynamics and cellular metabolism require thorough investigation [34]. Preclinical trials and long-term studies are essential to evaluate the durability of rejuvenation effects and to ensure that any risks, such as loss of cellular control or immunogenic reactions, are mitigated [35,36]. Establishing robust preclinical safety protocols and biomarkers for adverse effects will be essential before these therapies are widely implemented in clinical settings.

Stem cell exhaustion is a major hallmark of aging, as the body’s natural pool of stem cells, responsible for tissue repair and regeneration, declines over time [37,38]. This depletion reduces tissue repair, causing age-related degeneration in skin, muscle, and bone. Stem cell-based approaches aim to replenish aged cells or boost endogenous stem cell function to restore regeneration [39,40]. Stem cell transplants using young or genetically modified stem cells have shown potential in regenerating damaged tissues in preclinical studies. Endogenous activation rejuvenates stem cells using niche signals, growth factors, or extracellular vesicles. For instance, mesenchymal stem cells (MSCs) are used in various therapies to regenerate tissues, reduce inflammation, and enhance healing [41]. Both stem cell transplants and endogenous stem cell activation are promising avenues for rejuvenating aging tissues and restoring organ function [42,43].

Advances in gene-editing technologies, such as CRISPR-Cas9, have enabled precise modifications of genes involved in aging, offering a powerful tool for cellular rejuvenation [44]. Gene therapy allows scientists to repair or replace faulty genes that contribute to cellular dysfunction, or to upregulate genes associated with youthful cell function and longevity [45]. For example, reactivating telomerase, an enzyme that extends telomeres (protective caps on chromosomes that shorten with age), has been shown to delay cellular senescence and improve tissue function in aging cells.

In addition to gene therapy, protein therapy involves administering specific proteins that can rejuvenate cells and delay aging. For instance, the delivery of telomerase protein directly to cells can restore telomere length, protecting chromosomes from degradation and delaying cellular senescence [46,47]. Other therapeutic proteins, such as Klotho and sirtuins, have also demonstrated anti-aging effects by enhancing cellular repair mechanisms, improving mitochondrial function, and modulating inflammation [48]. Gene and protein therapies target those aging pathways to extend cellular and tissue health.

## 2. Mitochondrial Rejuvenation

Mitochondria, known as the cell’s powerhouses, play a crucial role in energy production and cellular health. However, with age, mitochondrial function declines due to accumulated damage in mitochondrial DNA (mtDNA), increased oxidative stress, and the dysregulation of mitochondrial dynamics, which disrupts the balance between mitochondrial fission and fusion [49,50]. This decline in mitochondrial function contributes to lower cellular energy, increased production of reactive oxygen species (ROS), and impaired metabolic efficiency, all hallmarks of aging.

Mitochondrial rejuvenation strategies aim to restore mitochondrial health and functionality, addressing the age-related decline in cellular energy production and resilience. These approaches focus on enhancing mitochondrial biogenesis, repairing damaged mitochondrial DNA (mtDNA), and reducing oxidative stress are three interdependent processes that are critical for maintaining mitochondrial integrity and function [37,51]. Therapeutics that activate mitochondrial biogenesis pathways, such as PGC-1α activators, or those that stabilize mtDNA, have shown promise in restoring mitochondrial function and extending cellular lifespan [52]. Additionally, experimental compounds targeting mitochondrial fission proteins (e.g., Drp1) [53] or fusion proteins (e.g., MFN1, MFN2, and OPA1) [54,55] help maintain healthy mitochondrial morphology and function, reducing oxidative damage and restoring cellular energy production.

A key pillar of mitochondrial rejuvenation is enhancing mitochondrial biogenesis, the process by which new mitochondria are created within cells. Therapeutics targeting biogenesis pathways, such as PGC-1α activators, play a vital role in increasing mitochondrial numbers and improving their quality. This enhancement contributes to better cellular energy metabolism and extended lifespan. Promising interventions, including dietary strategies like caloric restriction mimetics and pharmacological agents such as resveratrol and NAD+ precursors [56,57], are being explored to stimulate mitochondrial production and functionality, paving the way for non-invasive rejuvenation therapies. Equally important is the repair of mitochondrial DNA, which is particularly vulnerable to damage due to its exposure to reactive oxygen species (ROS) and limited repair capacity. Advances in precision medicine, such as CRISPR-Cas9 and mitochondrial-specific base-editing tools, provide novel means to correct mtDNA mutations [58]. These technologies stabilize the mitochondrial genome, preventing the cellular dysfunction and metabolic decline associated with accumulated mtDNA damage. By safeguarding mtDNA, these interventions help preserve energy production and reduce the risk of cascading cellular damage.

Another critical target of rejuvenation efforts is reducing oxidative stress, a hallmark of mitochondrial dysfunction and aging [59]. Excessive ROS generated by dysfunctional mitochondria damages proteins, lipids, and DNA, accelerating cellular aging [60]. Strategies for mitigating oxidative stress focus on boosting mitochondrial antioxidant defenses through compounds like mitoquinone (MitoQ) and SkQ1, which selectively target ROS within mitochondria [61,62]. Furthermore, optimizing mitochondrial dynamics through a balanced cycle of fission and fusion is essential for maintaining mitochondrial health. Proteins like Drp1 (fission) and MFN1, MFN2, and OPA1 (fusion) are pivotal in ensuring healthy mitochondrial morphology and mitigating oxidative damage [55,63].

Despite these advancements, the effective delivery of mitochondrial rejuvenation therapies remains a significant challenge (Table 1). Therapies targeting specific cell types, tissues, or subcellular structures must achieve precise localization to avoid unintended effects on healthy tissues. Emerging delivery technologies, such as mitochondrial-targeted nanoparticles and engineered stem cell therapies, show promise in addressing these obstacles, enabling localized and efficient therapeutic interventions.

The effective delivery of rejuvenation therapies is a significant obstacle to clinical translation, as most therapies need to target specific cell types, tissues, or even subcellular structures. For example, gene-editing therapies for mitochondrial rejuvenation or stem cell-based therapies for tissue regeneration must be delivered accurately to affected cells without affecting healthy tissues [64].

## 3. Challenges in Translating Cellular Rejuvenation to Clinical Applications

Despite the transformative potential of cellular rejuvenation therapies, translating these promising treatments from laboratory settings to clinical applications involves numerous challenges. While advances in cellular biology and biotechnology have laid a foundation for these therapies, ensuring their safety, efficacy, accessibility, and ethical application in human populations remains a complex task [65]. Key challenges include safety and efficacy concerns, effective delivery mechanisms, regulatory and ethical hurdles, and affordability and accessibility [66,67].

Safety is the foremost consideration in any new therapy, and cellular rejuvenation is no exception. Many rejuvenation techniques, such as epigenetic reprogramming and gene editing, involve manipulating fundamental cellular processes, which can inadvertently cause adverse effects, such as uncontrolled cell growth or tumor formation [68,69]. In particular, reprogramming cells to a more youthful state risks disrupting cellular identity and could potentially lead to the reactivation of oncogenes, raising the risk of cancer [35].

**Table 1:** A summary of drug delivery methods for rejuvenation therapies.

Delivery Method	Description	Advantages	Limitations	Ref.
Viral Vectors	Use modified viruses to deliver genetic material directly to targeted cells, commonly used in gene-editing therapies for rejuvenation.	High delivery efficiency; strong gene-editing capabilities.	Risk of immune response; potential off-target effects; concerns over long-term safety and integration stability.	[70,71]
Nanoparticles	Synthetic particles designed to carry therapeutic agents, including drugs or genetic material, to specific cells and tissues.	Low immunogenicity; customizable surface properties.	Limited targeting precision; challenges in crossing biological barriers, like the blood–brain barrier.	[72,73]
Liposomes	Lipid-based vesicles encapsulating therapeutic agents, protecting them from degradation until reaching targeted cells, commonly used for drug and gene delivery.	Low immunogenicity; protects cargo until delivery.	Limited targeting specificity; potential difficulty penetrating certain biological barriers.	[74]
CRISPR-Cas9 with Tissue-Specific Promoters	Modified CRISPR-Cas9 gene-editing system with tissue-specific promoters for increased precision, activating only in selected tissues for rejuvenation purposes.	High targeting precision; minimizes off-target risks.	Complex delivery requirements; limited by current research; risk of immune response with repeat dosing.	[45]
Biodegradable Nanoparticles	Advanced nanoparticles designed to degrade after delivery, minimizing toxicity and long-term side effects.	High biocompatibility; reduced long-term risk.	Developing precise targeting mechanisms remains a challenge; including stability issues in complex environments.	[75,76]
Engineered Extracellular Vesicles	Naturally derived vesicles engineered to carry therapeutic agents directly to aging cells or mitochondria, offering targeted rejuvenation effects.	High cellular compatibility; can naturally target cells.	Production scalability; limited payload capacity; precise targeting strategies are still in early stages of research.	[77,78]

Table 1 summarizes various delivery methods for rejuvenation therapies, highlighting each method’s unique strengths and limitations in clinical application. Traditional delivery mechanisms, including viral vectors, nanoparticles, and liposomes, each have advantages but come with limitations (Table 1). Viral vectors, while highly efficient, can trigger immune responses and pose risks of off-target effects. Nanoparticles and liposomes are less immunogenic but may lack sufficient targeting precision or struggle to penetrate biological barriers like the blood–brain barrier.

Recent advancements aim to develop non-invasive and highly targeted delivery systems to address these limitations. Innovations in nanotechnology, synthetic biology, and CRISPR-based delivery techniques are being explored to enable more controlled, tissue-specific, and even subcellular targeting [45]. For instance, CRISPR-Cas9 systems tailored with tissue-specific promoters could enhance the precision of gene-editing applications in rejuvenation therapies [79,80]. Additionally, research into biodegradable nanoparticles and engineered extracellular vesicles may provide more flexible and safer options for delivering rejuvenation therapies directly to aging cells or mitochondria, expanding the scope of potential treatments.

Effective delivery methods are at the heart of successful rejuvenation therapies, enabling precise targeting while minimizing adverse effects. Recent studies have highlighted innovative approaches that enhance therapeutic outcomes in aging-related interventions. For instance, Horvath et al. demonstrated the use of viral vectors to deliver Yamanaka factors (OSKM) to the hippocampus of aged rats, resulting in improved cognitive performance and partial epigenetic rejuvenation [70]. This study underscores the potential of viral-mediated gene delivery, particularly in brain tissues where precision and safety are paramount. Similarly, Lehmann et al. developed a regulatable adenovector system with a Tet-Off promoter, allowing controlled and reversible expression of OSKM genes [81]. This innovation minimizes risks associated with prolonged gene expression, providing a safer framework for cellular reprogramming therapies.

Adding to this, Jaijyan et al. explored the use of cytomegalovirus (CMV) vectors to deliver telomerase reverse transcriptase (TERT) and follistatin (FST) genes [82]. This approach, tested through both intranasal and intraperitoneal administration, extended lifespans in mice by up to 41% without carcinogenicity. The dual delivery routes demonstrated significant versatility, with intranasal delivery offering non-invasive access to the brain and injectable methods achieving systemic effects.

Extracellular vesicles (EVs) have emerged as a promising noncellular delivery platform, particularly for precision targeting. Ghosh et al. introduced engineered EVs (Exo-pep-11) conjugated with peptides targeting EphA4 receptors on neural stem cells (NSCs) [77]. This receptor-specific strategy significantly enhanced NSC proliferation and neurogenesis in aging rats, showcasing the potential of EVs for targeted therapies, especially in the nervous system where traditional delivery methods face challenges such as crossing the blood–brain barrier. Stem cell-derived EVs (SC-EVs) further highlight the versatility of this technology. Rather et al. reported that SC-EVs can successfully deliver therapeutic molecules like neprilysin to the brain via nasal administration, effectively addressing neurodegenerative conditions while bypassing systemic delivery challenges [83].

Natural compounds paired with nanoparticles are also gaining attention for their delivery efficiency and therapeutic benefits. Radwan et al. utilized a green synthesis approach to combine Eucalyptus camaldulensis bark extract with silver nanoparticles, enhancing the bioavailability of the extract [84]. This combination effectively inhibited cellular senescence and activated telomerase, highlighting the potential of nanoparticles as carriers that enhance the stability and efficacy of bioactive compounds.

Despite these advancements, challenges remain in optimizing delivery systems for clinical applications. Issues such as scalability, biodistribution, immune responses, and potential off-target effects need to be addressed. Viral vectors, while precise, may elicit immune reactions or pose integration risks, while extracellular vesicles require rigorous quality control and optimization for large-scale production. Addressing these limitations will be essential for the broader application of these technologies.

Future directions may involve hybrid strategies, such as combining EV-based delivery with gene-editing tools like CRISPR-Cas9 or integrating nanoparticles with bioactive compounds for improved targeting and therapeutic stability. Advances in nanotechnology, biomaterials, and bioengineering are expected to drive these innovations, making therapies safer, more effective, and accessible to diverse populations.

The regulatory landscape for cellular rejuvenation therapies is complex, as these treatments often operate at the edge of biotechnology, encompassing areas like gene editing, stem cell therapy, and synthetic biology [85]. Regulatory agencies, such as the U.S. Food and Drug Administration (FDA), the European Medicines Agency (EMA), and other international bodies, require extensive proof of safety and efficacy through rigorous clinical trials [86]. However, due to the novelty of rejuvenation therapies, regulatory pathways are not fully established, which can delay approval and increase costs [87]. Regulatory agencies must adapt to accommodate the unique mechanisms of action and risk profiles associated with rejuvenation therapies.

Furthermore, rejuvenation therapies raise significant ethical concerns, particularly regarding long-term health impacts, potential germline modifications, and accessibility [88,89]. Rejuvenation therapies hold immense potential to improve health and extend lifespan, but they also raise profound ethical concerns that must be carefully addressed to ensure responsible development and equitable access. Key issues include long-term health impacts, the ethical implications of germline modifications, and the risk of exacerbating societal inequalities.

Gene-editing therapies, particularly those involving tools like CRISPR-Cas9, introduce genetic changes that could be passed to future generations through germline modifications. While this approach holds promise for preventing hereditary diseases, it also raises significant ethical concerns about unintended consequences, such as unforeseen genetic complications or ecological impacts on the human gene pool. Germline editing challenges societal notions of consent, as future generations cannot agree to modifications that could permanently alter their genetic makeup. Additionally, these interventions could lead to genetic stratification, where enhancements are accessible only to those with financial means, further entrenching societal inequities.

Accessibility is another critical issue. If rejuvenation therapies remain prohibitively expensive, they risk becoming exclusive to affluent individuals or nations, deepening global health disparities. Such inequities could lead to a “longevity divide”, where some populations experience dramatically extended health spans while others continue to face preventable age-related illnesses. This disparity has the potential to exacerbate existing socio-economic tensions, creating ethical dilemmas about the right to health and equitable access to medical advancements (Table 2).

The long-term health impacts of rejuvenation therapies, particularly gene-editing and cellular reprogramming, are another area of concern. While these therapies aim to restore youthful function, their effects on aging populations over decades remain uncertain. Risks such as off-target genetic effects, immune reactions, or the potential emergence of novel age-related conditions necessitate comprehensive long-term studies before widespread clinical use. Ensuring that rejuvenation therapies are not only effective but also accessible is a key hurdle to overcome. Many cellular rejuvenation therapies involve high research and development costs, complex manufacturing processes, and sophisticated delivery systems, contributing to high prices that may place these therapies out of reach for most individuals.

## 4. CRISPR Technology for Rejuvenation

CRISPR-Cas9, one of the most powerful gene-editing tools available, has become a cornerstone of rejuvenation research due to its precision, ease of use, and versatility [45]. The CRISPR system works by utilizing a guide RNA (gRNA) to direct the Cas9 enzyme to a specific DNA sequence, where it can introduce targeted cuts or edits [90]. This technology allows scientists to modify genes associated with aging and cellular dysfunction with high precision, which is critical for rejuvenation therapies.

**Figure 1:**
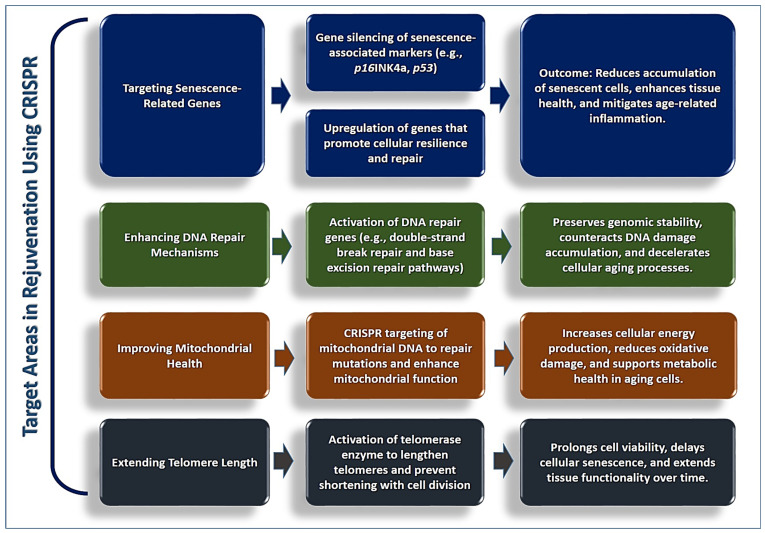
A detailed structure that highlights each rejuvenation target using CRISPR.

Cellular senescence, where cells lose their ability to divide and adopt a pro-inflammatory state, is a major contributor to tissue aging. By using CRISPR to silence genes associated with senescence or to upregulate genes that enhance cellular resilience, researchers can reduce the number of senescent cells and promote tissue health [91]. For example, silencing the p16INK4a or p53 pathways, which are involved in the onset of cellular senescence, could help delay or reverse aging effects in targeted tissues [92,93]. However, it must be noted that the use of gene-editing technology like CRISPR and the production and distribution of personalized stem cell treatments are inherently expensive processes that require specialized facilities and expertise. Figure 1 shows a refined structure that highlights each rejuvenation target, specific gene-editing interventions, and the direct benefits to cellular health and aging.

Age-related decline in DNA repair capacity contributes to genomic instability, a hallmark of aging. CRISPR can be used to upregulate genes involved in DNA repair pathways, such as those governing the repair of double-strand breaks or base excision repair [94,95]. By enhancing these pathways, cells can maintain genomic stability, thus slowing down the aging process and reducing the risk of age-related diseases like cancer [96].

Mitochondria, the cell’s energy powerhouses, play a key role in aging. Recently, CRISPR-based systems targeting mitochondrial DNA have emerged, enabling scientists to repair mutations and enhance mitochondrial function [91,97]. Since mitochondrial dysfunction is associated with reduced energy production and increased oxidative stress, targeting mitochondrial genes could significantly improve cellular health in aging tissues.

Telomeres, the protective caps at the ends of chromosomes, shorten with each cell division, eventually leading to cell aging. CRISPR could be used to activate telomerase, the enzyme responsible for elongating telomeres, thus extending the lifespan of cells and delaying the onset of cellular senescence (Figure 1).

Figure 1 visually represents CRISPR interventions targeting key aging mechanisms such as cellular senescence, DNA repair pathways, mitochondrial function, and telomere maintenance, reinforcing the narrative of how these therapies address aging at multiple levels. For instance, targeting genes like p16INK4a or p53 to suppress senescence pathways aligns with the emphasis on reducing the pro-inflammatory effects of senescent cells and promoting tissue health. Similarly, the connection between CRISPR and enhanced DNA repair pathways, such as BRCA1 or XRCC1, illustrates how genomic stability can be maintained to mitigate age-related diseases like cancer. The figure also highlights CRISPR’s potential to improve mitochondrial function by editing mitochondrial DNA (mtDNA), reducing oxidative stress, and boosting cellular energy production, which are are key points discussed in the text. Furthermore, CRISPR-mediated activation of telomerase addresses telomere shortening, extending cellular lifespan and delaying senescence.

Ethical and safety challenges, such as off-target effects and heritable germline modifications, are also visualized in the figure. These complement the focus on refining CRISPR specificity with advanced tools like base editors and prime editors, ensuring safer applications. It underscores the importance of balancing therapeutic potential with the need for stringent ethical and regulatory oversight.

While gene editing offers enormous potential, it also poses significant challenges [98,99]. There is the risk of off-target effects, where CRISPR inadvertently edits unintended parts of the genome, potentially leading to adverse effects such as cancer. Furthermore, because some gene-editing changes could be heritable if applied to germline cells, ethical concerns arise regarding the potential for unintended impacts on future generations. Ongoing research has been focused on refining CRISPR’s specificity and developing advanced gene-editing tools like base editors and prime editors to make rejuvenation therapies safer and more effective [100].

To make these therapies accessible on a global scale, strategies must be implemented to address their cost (Table 3). Government subsidies and public–private partnerships could support clinical trials and lower development costs. Additionally, efforts in scalable manufacturing and standardized production protocols can help reduce prices over time.

For instance, advances in bio-manufacturing, such as the use of bioreactors for large-scale production of stem cells or engineered proteins, could bring down production costs [101]. Recently, Lee at al. reported on the development of a large-scale smart bioreactor system featuring fully integrated wireless membrane sensors and advanced electronics for continuous, long-term, in situ monitoring of stem cell cultures. This innovative technology uses low-profile, label-free nano-membrane sensors and ultrathin electronics, which seamlessly integrate with commercial cell culture bags. This setup enables in-line culture monitoring and delivers real-time feedback, optimizing the culture environment and enhancing process efficiency [102].

Furthermore, research into generic or universal therapies, such as off-the-shelf stem cell therapies or widely applicable senolytics could reduce the need for individualized treatments, enhancing affordability and accessibility. By prioritizing cost-effective solutions and exploring subsidy and funding options, rejuvenation therapies may become viable healthcare options for a broader population.

## 5. Personalized Stem Cell Treatments for Rejuvenation Therapies

Stem cells offer transformative potential in rejuvenation therapies due to their unique ability to differentiate into various cell types, self-renew, and regenerate damaged tissues. Unlike gene editing, which alters gene expression at a molecular level, stem cell therapies offer a cellular approach to rejuvenation by replenishing the body’s natural regenerative capacity. Personalized stem cell treatments involve isolating stem cells from the patient, expanding them in controlled laboratory environments, and then reintroducing them to the body to target specific tissues in need of repair or rejuvenation. This autologous (self-sourced) approach minimizes the risk of immune rejection, making it a powerful tool in personalized medicine. It encompasses the use of induced pluripotent stem cells (iPSCs) and mesenchymal stem cells for rejuvenation therapy (Table 2).

Induced pluripotent stem cells (iPSCs) are generated by reprogramming a patient’s somatic cells, such as skin or blood cells, to revert them to a pluripotent state, meaning they have the ability to become any cell type in the body. This breakthrough approach allows for the creation of patient-specific cells tailored for transplantation, minimizing immune rejection. iPSCs are a cornerstone in rejuvenation therapy because they provide a versatile source for regenerating a range of tissues.

For instance, if a patient suffers from age-related heart degeneration, iPSCs can be directed to differentiate into cardiomyocytes, which can then be transplanted to repair or replace damaged heart tissue, enhancing heart function and potentially reversing damage [103,104]. iPSCs are also being explored for neurodegenerative diseases, such as Parkinson’s disease, where they can be programmed to become dopaminergic neurons and support brain health. However, iPSCs must be carefully screened before reintroduction to eliminate undifferentiated cells, which could potentially form tumors, adding complexity to the process (Table 4).

Mesenchymal stem cells (MSCs) are multipotent stem cells that can differentiate into several tissue types, including bone, cartilage, and fat cells (Table 2). Known for their anti-inflammatory and immunomodulatory properties, MSCs are increasingly used in anti-aging and regenerative therapies [105]. MSCs can be sourced from the patient’s own tissues, such as bone marrow or adipose tissue, which makes them ideal for autologous therapies, reducing the risk of immune rejection and enhancing safety. MSC therapies have shown considerable promise in treating conditions associated with aging, such as osteoarthritis, sarcopenia (age-related muscle loss), and degenerative disc disease [106,107,108]. In these applications, MSCs work by secreting bioactive molecules that modulate inflammation, encourage tissue repair, and recruit endogenous cells to aid in regeneration. The versatility and broad therapeutic potential of MSCs make them one of the most accessible and widely researched stem cells in rejuvenation therapies.

Apart from iPSCs and MSC therapies, exosomes have also shown a great promise for the for rejuvenation therapy [109,110]. Exosomes are small vesicles secreted by stem cells that contain various bioactive compounds, including proteins, lipids, and RNA. These vesicles act as “biological messengers”, delivering rejuvenating signals to targeted cells and tissues without the need for full stem cell transplantation. Stem cell-derived exosomes can influence cell behavior, promote cellular repair, and reduce inflammation, making them a promising alternative for those who may not be candidates for direct stem cell therapy [66,111].

One significant advantage of exosome therapy is its relative simplicity compared to cell transplantation. Exosomes are easier to store, transport, and administer, potentially lowering logistical and regulatory barriers and making the therapy more scalable [112,113]. Moreover, because they are cell-free, exosomes do not carry the risk of forming unwanted cell growth or tumors. Research is ongoing to harness exosomes for rejuvenation therapies, with promising applications in skin rejuvenation, joint repair, and even neurodegenerative diseases [42,110].

Gene-editing technologies, such as CRISPR-Cas9, have opened new avenues in stem cell therapies by allowing scientists to enhance the rejuvenative potential of stem cells. By combining gene editing with stem cell therapy, researchers can modify the genetic code of induced pluripotent stem cells (iPSCs) or mesenchymal stem cells (MSCs) to increase their efficacy in regenerative applications. For instance, iPSCs can be edited to upregulate anti-aging genes or enhance telomerase activity, potentially delaying cellular senescence and improving their regenerative capacity [114]. In MSCs, gene editing can boost resilience against oxidative stress or increase the secretion of therapeutic factors, making these cells more effective for conditions like inflammatory joint diseases and age-related tissue degeneration [115]. Moreover, gene-edited stem cells thus offer a powerful dual approach: they combine the intrinsic regenerative abilities of stem cells with the precision of gene-editing to target age-related dysfunctions at both cellular and genetic levels.

Despite the high promise, the production and distribution of personalized stem cell treatments involve a series of technical, regulatory, and logistical challenges. Personalized stem cell therapies are inherently costly, as each patient’s cells must be individually processed. Large-scale production requires specialized facilities, highly trained personnel, and strict quality controls to ensure that the cells meet clinical-grade standards. Expanding these treatments to a larger population would require innovations in scalable manufacturing, such as the use of bioreactors to grow cells at high volumes, while still maintaining safety and efficacy.

Moreover, stem cells exhibit variability in behavior, potency, and therapeutic effect based on their source, processing methods, and culture conditions. This variability poses challenges in standardizing stem cell products and ensuring consistent results across patients. Rigorous quality control and validation protocols must be in place to ensure that each batch of stem cells meets safety and potency standards before it is used therapeutically.

In addition, stem cell therapies are subject to stringent regulatory oversight due to their potential risks, such as immune reactions, unintended differentiation, and tumor formation. Regulatory agencies like the U.S. FDA and EMA require extensive preclinical and clinical testing to validate the safety and efficacy of these treatments [116]. Additionally, because stem cell therapies often fall under advanced therapy medicinal products (ATMPs), they require compliance with specific guidelines, including traceability, safety testing, and monitoring of adverse effects [117].

The autologous nature of personalized stem cell therapies makes distribution complex. Cells must often be processed and delivered quickly to maintain viability, and specialized cold-chain logistics may be needed to preserve cell quality during transport [118,119]. The development of centralized production hubs and streamlined supply chains will be essential to make these therapies more accessible on a large scale.

Currently, personalized stem cell treatments are often expensive and may not be covered by insurance, limiting their accessibility to only a few. Public and private partnerships, as well as government subsidies and funding initiatives, will be essential to make these therapies more affordable and accessible. In the future, innovations in automated manufacturing and delivery processes could reduce costs, making stem cell rejuvenation therapies a feasible option for a broader demographic.

## 6. Future Directions and the Path to Clinical Success

Despite the transformative potential of cellular rejuvenation therapies, translating these promising treatments from laboratory settings to clinical applications involves numerous challenges. While advances in cellular biology and biotechnology have laid a foundation for these therapies, ensuring their safety, efficacy, accessibility, and ethical application in human populations remains a complex task. Key challenges include safety and efficacy concerns, effective delivery mechanisms, regulatory and ethical hurdles, and affordability and accessibility [120].

Widespread clinical adoption of cellular rejuvenation therapies could transform how age-related diseases and conditions are treated, potentially extending the healthy lifespan of millions worldwide. Achieving this, however, will require a coordinated effort across scientific, medical, regulatory, and public sectors. Translating the promise of cellular rejuvenation into accessible therapies calls for innovative strategies that ensure safety, efficacy, affordability, and ethical alignment [32,39]. Key pathways to accelerate this transition include innovative research collaborations, improved biomarkers, personalized approaches, robust ethical frameworks, and scalable, affordable solutions.

The complexity of cellular rejuvenation demands insights from multiple scientific disciplines. Establishing interdisciplinary research initiatives that bring together molecular biologists, bioengineers, data scientists, clinicians, and experts in artificial intelligence will be critical [40,121]. For example, integrating bioinformatics with molecular biology can help map the effects of cellular rejuvenation therapies on gene expression and protein networks [122,123]. Cross-institutional partnerships between academic institutions, biotech companies, and hospitals can also facilitate shared access to resources, data, and funding, streamlining the development and testing of therapies.

Collaborations with patient advocacy groups and healthcare providers ensure that research meets real-world needs, refines therapeutic goals, and accelerates clinical acceptance. International partnerships are also crucial for knowledge sharing and harmonizing protocols across healthcare systems.

Effective biomarkers are vital for monitoring the success of rejuvenation therapies, providing quantifiable indicators of cellular and physiological responses to treatment [32]. Biomarkers that reflect mitochondrial health, epigenetic changes, cellular senescence, and regenerative capacity will be especially valuable for evaluating therapeutic efficacy and safety in clinical trials (Table 5). Current biomarkers for age-related changes, such as DNA methylation clocks or markers of mitochondrial function, serve as starting points, but more sophisticated and specific markers are needed to capture the nuances of cellular rejuvenation [124,125].

Developing minimally invasive biomarkers, such as blood-based markers for senescent cell clearance or rejuvenation proteins, can streamline clinical trials and improve patient compliance. Imaging biomarkers, like fluorescence-based mitochondrial assessments, can provide real-time data on therapeutic effects [126,127]. These markers would also be essential for post-treatment monitoring, allowing clinicians to track long-term outcomes and detect potential adverse effects early.

A personalized approach to cellular rejuvenation, considering genetic, metabolic, and lifestyle factors, is likely more effective than a generalized model. It would optimize treatment based on genetic predispositions, age-related changes, and health history.

Advances in artificial intelligence (AI) and genetic profiling can facilitate this personalized approach. For example, AI algorithms can analyze large-scale genomic and epigenomic data to identify patients who may benefit most from specific rejuvenation therapies [128,129]. Genomic and transcriptomic profiling could reveal patient-specific markers that predict responsiveness to treatments, allowing for customized dosing regimens and therapeutic adjustments [130]. Personalized therapies would also help minimize risks by tailoring treatments to individual biological profiles, reducing the likelihood of adverse responses. To make personalized rejuvenation therapies viable, adaptable clinical workflows and treatment protocols must integrate genetic and phenotypic data. This will require technological advances and extensive clinician education to manage and interpret patient-specific data.

As cellular rejuvenation therapies move closer to clinical application, clear ethical and regulatory frameworks are essential for guiding their development and use [85]. These frameworks should address key ethical issues, such as the long-term effects of gene-editing therapies, the potential for unequal access, and the societal implications of extended lifespan. For instance, gene editing and stem cell-based rejuvenation treatments raise concerns about potential germline modifications or unintended hereditary effects, which require robust oversight. Transparent regulatory guidelines can help mitigate public concerns, build trust, and prevent the misuse of these therapies [131].

For cellular rejuvenation therapies to have a meaningful impact, they must be affordable and accessible. High production costs, complex R&D, and advanced delivery systems currently drive up prices. Addressing this requires scalable manufacturing, streamlined logistics, and public–private support. Standardizing protocols, such as batch manufacturing for stem cells or scalable gene-editing synthesis, can reduce costs. Developing generic treatments, like off-the-shelf stem cells or broad senolytics, could further improve affordability.

Cellular rejuvenation therapies hold great promise in transforming healthcare by addressing aging’s root causes and extending lifespan. Achieving this requires a multidisciplinary approach combining scientific rigor, ethical integrity, regulatory foresight, and accessibility. Through collaborative research, advanced biomarkers, personalized treatments, ethical governance, and scalable production, these therapies can transition from experimental success to clinical reality, ushering in an era focused on health preservation and restoration.

## 7. Artificial Intelligence and Machine Learning for Cellular Rejuvenation

Artificial intelligence (AI) and machine learning (ML) are revolutionizing cellular rejuvenation research, offering tools to accelerate discoveries, improve precision, and enhance treatment strategies [132]. By leveraging vast datasets, AI provides unprecedented capabilities for predicting treatment outcomes, designing clinical trials, and tailoring personalized therapies, making it a cornerstone of next-generation rejuvenation research [133]. AI’s predictive modeling capabilities are particularly valuable in understanding the complex biological processes underlying cellular aging and rejuvenation [134]. Machine learning algorithms can analyze high-dimensional datasets, such as genomic, transcriptomic, and proteomic profiles, to identify key biomarkers associated with aging and therapeutic efficacy. These insights not only refine the selection of targets for interventions like CRISPR-Cas9 or senolytic therapies but also predict patient-specific responses, reducing trial-and-error approaches and increasing treatment success rates.

In clinical trial design, AI is reshaping the landscape by enabling adaptive trials that are more efficient and cost-effective. By simulating diverse trial scenarios, AI can identify optimal participant groups, predict potential adverse effects, and assess the likelihood of therapeutic success before trials commence [135,136]. This reduces the risk of costly failures and accelerates the translation of cellular rejuvenation therapies from bench to bedside.

Personalized therapy strategies are another area where AI is making significant strides. By integrating patient-specific data, including genetic, epigenetic, and lifestyle factors, AI models can design tailored interventions that maximize efficacy while minimizing risks. For example, AI-driven algorithms can predict the most effective combination of gene-editing targets or senomorphic treatments for an individual, enabling a precision medicine approach to rejuvenation [137].

Furthermore, AI aids in optimizing the delivery of cellular rejuvenation therapies [137]. Machine learning models can refine delivery systems, such as nanoparticles or extracellular vesicles, by predicting biodistribution and identifying factors that enhance targeting specificity. This ensures that therapies reach their intended cellular targets with minimal off-target effects, improving both safety and efficiency.

AI also plays a crucial role in the long-term monitoring and evaluation of rejuvenation therapies. By analyzing real-time patient data from wearable devices and other sensors, AI systems can detect early signs of adverse effects or therapy resistance, allowing for timely interventions. This proactive approach enhances the safety profile of emerging therapies and builds confidence in their clinical applications.

While the integration of AI into rejuvenation research holds immense promise, it also raises challenges, including data privacy concerns, the need for robust validation of AI models, and ensuring equitable access to AI-driven technologies. Addressing these issues through transparent regulatory frameworks and interdisciplinary collaboration will be essential for the responsible development of AI in this field.

Incorporating AI and ML into cellular rejuvenation research not only accelerates the pace of innovation but also makes therapies more precise, effective, and accessible. By harnessing the power of AI, the field is poised to move toward a future where cellular rejuvenation is not only a scientific aspiration but also a transformative reality in medicine.

## 8. Inflammation Determinants as Major Targets for Therapy of Senescence-Associated Disorders

Chronic inflammation is a hallmark of aging and plays a critical role in the development of various age-related diseases, including neurodegenerative disorders, cardiovascular diseases, and osteoarthritis [138,139]. This inflammation, often referred to as inflammation, is driven by a complex interplay of genetic, cellular, and environmental factors. Among the key determinants of inflammation are genes that code for pro-inflammatory molecules as well as the types of inflammatory cells involved. These factors provide promising targets for therapeutic interventions aimed at alleviating senescence-associated conditions and improving health span.

One of the main contributors to the inflammatory response in senescence is the upregulation of pro-inflammatory cytokines and chemokines, which are often encoded by specific genes. Notably, genes like TNF-α (tumor necrosis factor-alpha), IL-6 (interleukin-6), IL-1β (interleukin-1 beta), and CRP (C-reactive protein) are key regulators of inflammation in aging tissues [140]. These genes are often activated in response to cellular stress, oxidative damage, and senescence, and their overexpression is linked to chronic, low-grade inflammation. For example, TNF-α and IL-6 are involved in amplifying the inflammatory cascade through the activation of nuclear factor kappa B (NF-κB), a critical regulator of immune response and cellular stress [141,142]. Overactive NF-κB signaling has been observed in aged tissues and senescent cells, leading to sustained inflammation that contributes to tissue dysfunction and the acceleration of age-related diseases. Targeting these pro-inflammatory cytokine genes offers a direct therapeutic strategy to reduce inflammation in senescent tissues and limit its pathological effects [143,144,145].

In addition to the genetic regulation of inflammation, the accumulation of specific inflammatory cell types is a central feature of senescence. These cells, particularly senescent immune cells, contribute to the chronic inflammatory environment. One key group involved is senescent macrophages, which are known to secrete high levels of pro-inflammatory cytokines, perpetuating the inflammatory milieu. Macrophages are part of the innate immune system and play a crucial role in tissue repair and immune surveillance [146,147]. However, as they age or become senescent, their function becomes dysregulated, shifting from tissue repair to the promotion of inflammation.

Similarly, T cells, particularly CD8+ T cells, have been implicated in age-related inflammatory response. In aging individuals, the number of senescent T cells increases, and these cells contribute to the maintenance of chronic inflammation. This accumulation of senescent T cells can impair immune responses, making the body more susceptible to infections and age-related diseases. Another key player in inflammation is the neutrophil, a type of white blood cell that is often elevated in aged tissues. While neutrophils are essential for fighting infections, their overactivation in senescence leads to an excessive release of reactive oxygen species (ROS) and inflammatory mediators, contributing to tissue damage and aging.

Given the central role of inflammation in aging, several therapeutic strategies focus on modulating these determinants to alleviate senescence-associated conditions. Senolytic therapies, which aim to selectively eliminate senescent cells, have shown promise in reducing the burden of pro-inflammatory cells and cytokines. By targeting and clearing senescent cells, senolytics can reduce the overall inflammatory load and improve tissue function in aging organisms.

Another promising strategy is the inhibition of inflammatory cytokine signaling, particularly targeting key pathways such as NF-κB, JAK-STAT, and NLRP3 inflammasome activation [148]. Small molecules or biologics that inhibit these pathways could reduce the production of pro-inflammatory cytokines like TNF-α and IL-6, thus dampening the chronic inflammation that drives age-related diseases. For example, drugs like tocilizumab, an IL-6 receptor antagonist, have already been shown to have anti-inflammatory effects and are being tested for their potential to mitigate inflammation and associated diseases [149,150].

Additionally, immune checkpoint inhibitors that modulate the activity of senescent immune cells, such as senescent T cells, are an emerging area of interest. By rejuvenating the immune system and restoring the balance between pro- and anti-inflammatory responses, these therapies could reduce inflammation while enhancing immune surveillance.

While targeting inflammation determinants holds great promise, several challenges remain in translating these strategies into effective therapies. One challenge is the specificity of targeting pro-inflammatory molecules and cells without compromising normal immune responses, which are essential for tissue repair and defense against infections. The aging immune system is often in a delicate balance, and the overmodulation of inflammatory pathways could have unintended consequences, including increased susceptibility to infections and cancer. Moreover, the development of therapies that can effectively reach and act on senescent cells in specific tissues remains a key obstacle. Nano-medicine and targeted delivery systems may provide solutions by delivering anti-inflammatory agents directly to senescent cells or inflamed tissues.

To summarize, inflammation determinants, including pro-inflammatory genes and specific immune cell types, are critical drivers of senescence-associated conditions. Targeting these factors through gene editing, senolytic therapies, and immune modulation holds significant potential for treating aging-related diseases and improving health span. As research progresses, the development of targeted, safe, and effective strategies will be essential to harness the therapeutic potential of modulating inflammation in aging.

## 9. Conclusions

Cellular rejuvenation therapies hold transformative potential, shifting medicine from treating age-related diseases to proactively extending health span and promoting rejuvenation. While promising preclinical results inspire hope for managing, slowing, or even reversing aging, significant challenges remain on the path to clinical application.

Key concerns include ensuring safety and efficacy, as manipulating cellular mechanisms carries risks like uncontrolled cell proliferation. Delivery systems, such as viral vectors and nanoparticles, require refinement to enhance specificity and minimize impacts on healthy tissues. Regulatory and ethical hurdles further complicate progress, necessitating transparent frameworks to ensure safety, equitable access, and public trust. High costs also pose barriers, highlighting the need for scalable manufacturing, standardized protocols, and collaborative public–private initiatives to improve affordability.

Achieving widespread adoption will require coordinated efforts among researchers, healthcare providers, regulators, and policymakers. With continued innovation, interdisciplinary collaboration, and a commitment to ethical and accessible solutions, cellular rejuvenation therapies could redefine healthcare, offering individuals the possibility of healthier, longer lives.

## Figures and Tables

**Table 2:** Proposed solutions for ethical and societal concerns in rejuvenation therapies.

Concerns	Proposed Solutions	Details/Actions
Ethical Guidelines for Germline Modifications	Develop and enforce internationally agreed-upon ethical standards.	Restrict germline editing to severe hereditary conditions; prohibit non-health-related enhancements; involve global bodies like WHO and UNESCO for oversight.
Equitable Access	Establish policies ensuring therapies are accessible to all populations, regardless of income or geography.	Implement tiered pricing models, public–private partnerships, and global funding mechanisms to subsidize costs for low- and middle-income countries.
Long-Term Safety and Efficacy	Require rigorous research and monitoring for rejuvenation therapies before and after clinical approval.	Conduct extensive preclinical and clinical trials; establish long-term follow-ups to monitor safety; regulatory agencies to mandate robust safety and efficacy data.
Public Engagement and Education	Build public trust through transparent information and inclusive discussions on the risks, benefits, and implications of rejuvenation therapies.	Use public forums, educational campaigns, and stakeholder consultations; ensure diverse representation in policymaking to reflect societal values.
Policy and Legal Safeguards	Create comprehensive legal frameworks to regulate the development and application of therapies.	Define clear boundaries between therapeutic and enhancement uses; enforce consent and privacy protections; regulate against misuse of gene-editing technologies.
Global Collaboration	Foster international cooperation to ensure equitable global access to rejuvenation innovations.	Adapt models like COVAX for medical innovations; encourage technology transfer and shared research resources among nations; develop a global distribution plan for rejuvenation therapies.

**Table 3:** CRISPR technology for rejuvenation: risks, off-target effects, and ethical concerns.

Aspect	Description	Examples	Risks and Concerns
Mechanism of Action	Utilizes the Cas9 protein guided by RNA sequences to make precise cuts in DNA, enabling targeted gene editing.	Targeting genes regulating aging processes, such as FOXO3A or Klotho, to enhance longevity.	Risk of off-target effects, where unintended genetic regions are altered, potentially leading to harmful mutations.
Applications	Gene therapy that repairs age-related DNA damage, regulates senescence, or enhances mitochondrial function.	Editing mitochondrial-related genes like POLG to improve mtDNA stability.	Delivery challenges, requiring precise targeting to avoid effects on healthy tissues.
Advantages	High precision, versatility, and efficiency in targeting specific genes for age-related conditions.	Correction of progeria-related mutations to slow aging; regulation of inflammation-related genes.	Risk of immune responses to CRISPR components, which can reduce effectiveness or cause side effects.
Mitochondrial Targeting	Emerging strategies for editing mtDNA to correct mutations affecting energy metabolism and aging processes.	Techniques like mitoCRISPR to bypass limitations of nuclear DNA targeting.	Technical barriers to efficient mitochondrial editing due to unique structural features of mtDNA.
Ethical Concerns	Potential use in heritable (germline) modifications that could be passed to future generations.	Preventing the inheritance of age-related disorders like Huntington’s disease.	Raises concerns about eugenics, long-term unforeseen consequences, and lack of consent from future generations.
Safety Challenges	Ensuring the edited gene functions as intended without unintended secondary effects.	Testing CRISPR on genes regulating senescence without disrupting beneficial cellular processes.	Functional uncertainty, as gene modifications may have unpredictable or pleiotropic effects.
Regulatory Challenges	Strict approval processes and global variability in regulatory frameworks for gene editing.	Differences in laws governing somatic vs. germline editing across countries.	Slower clinical adoption due to the need for comprehensive trials and societal debates on ethics and safety.
Therapeutic Delivery	Developing systems to deliver CRISPR components to specific tissues or cells affected by aging.	Use of nanoparticles or viral vectors to deliver CRISPR tools to senescent cells or stem cells.	Risks of systemic exposure causing off-target effects or immune system activation.
Future Innovations	Advancements in base editing and prime editing for more precise and less invasive modifications.	Correcting single base mutations in age-related diseases like Alzheimer’s or Parkinson’s.	Ethical dilemmas in distinguishing therapeutic from enhancement applications.

**Table 4:** Comprehensive overview of personalized stem cell treatments for rejuvenation, highlighting key aspects, associated challenges, and possible solutions.

Aspect	Details	Problems	Possible Solutions
Stem Cell Potential in Rejuvenation	Stem cells can differentiate into various cell types, self-renew, and regenerate damaged tissues, offering a cellular approach to rejuvenation that enhances the body’s natural regenerative abilities.	High variability in cell behavior and potency, impacting therapeutic consistency.	Establish strict quality control and validation protocols to ensure consistent results.
Personalized Stem Cell Treatments	Involves isolating and expanding patient’s stem cells in a lab, then reintroducing them to target specific tissues. This autologous approach reduces immune rejection risks, using iPSCs and MSCs for targeted rejuvenation.	Expensive, time-consuming, and complex process requiring individualized cell preparation.	Develop automated and standardized processing techniques to reduce costs and time.
Induced Pluripotent Stem Cells (iPSCs)	iPSCs are created by reprogramming a patient’s somatic cells to a pluripotent state, allowing differentiation into any cell type. iPSCs are versatile, suitable for heart, neural, and other tissue repairs but require careful screening to avoid undifferentiated cells that could form tumors.	Risk of tumor formation from undifferentiated cells and complex screening processes.	Improve screening technologies and establish protocols to ensure complete differentiation before reintroduction.
Mesenchymal Stem Cells (MSCs)	MSCs are multipotent cells capable of differentiating into bone, cartilage, and fat cells, with strong anti-inflammatory and immunomodulatory properties. MSCs are used in treatments for aging conditions like osteoarthritis and sarcopenia.	Potential immune response or rejection if autologous sourcing is unavailable; limited differentiation range.	Increase research on MSCs’ differentiation abilities and explore allogeneic MSCs with minimized rejection risk.
Exosome Therapy	Exosomes are bioactive vesicles from stem cells that deliver rejuvenating signals to cells without stem cell transplantation. Exosome therapy is simpler and potentially safer than full-cell transplantation and shows promise in rejuvenation.	Less understood than full-cell therapies; requires extensive validation to ensure efficacy and safety.	Conduct rigorous research to establish reliable therapeutic protocols and efficacy data for clinical use.
Gene-Editing in Stem Cell Therapies	Gene-editing tools like CRISPR-Cas9 enhance stem cell rejuvenation potential by modifying genes in iPSCs or MSCs to upregulate anti-aging genes or increase resilience. This combination of gene editing and stem cell therapy targets aging at cellular and genetic levels.	Potential unintended genetic modifications and ethical concerns with gene editing.	Develop targeted gene-editing protocols with precise controls and maintain ethical transparency.
Challenges in Production and Distribution	High costs, specialized facilities, skilled personnel, and quality controls make large-scale stem cell therapy production complex. Scalable manufacturing solutions like bioreactors are needed to expand accessibility.	Expensive and resource-intensive process limits access and scalability.	Invest in automated bioreactor technology to scale up production and reduce costs for larger populations.
Stem Cell Variability and Standardization	Stem cells vary in behavior based on source and processing, complicating standardization across patients. Quality control and validation protocols are critical to ensure consistent safety and potency.	Difficulty achieving consistent quality and outcomes across patients due to variability.	Establish standardized processing protocols and conduct thorough testing of each batch to ensure reliability and safety.
Regulatory Challenges	Stem cell therapies require stringent oversight (e.g., by FDA and EMA) to manage risks like immune reactions and tumor formation. Compliance with ATMP guidelines, including traceability and safety testing, is mandatory.	Complex regulatory requirements add time and cost, potentially delaying therapy access for patients.	Work closely with regulatory agencies to streamline approval processes and develop standardized documentation and testing to meet compliance standards efficiently.
Distribution Logistics	Autologous therapies need rapid processing and specialized cold-chain logistics for maintaining cell viability. Centralized hubs and streamlined supply chains are essential for broader access.	Cold-chain logistics and rapid processing requirements increase costs and complexity.	Develop centralized production and processing hubs to streamline logistics and ensure rapid delivery, while investing in optimized cold-chain solutions.
Cost and Accessibility	Currently, high costs limit accessibility. Public–private partnerships, subsidies, and funding are needed to make these therapies affordable. Innovations in automated manufacturing and delivery could reduce costs for wider use.	High costs make therapies inaccessible to many, and insurance coverage is limited.	Secure government and private partnerships for subsidies and advance automation technologies to reduce manufacturing costs and improve accessibility.

**Table 5:** Comparison of rejuvenation approaches, covering iPSCs, MSCs, exosomes, and CRISPR.

Approach	Advantages	Limitations	Current Clinical Applications	Unique Challenges
Induced Pluripotent Stem Cells (iPSCs)	Potential to differentiate into any cell type; autologous use reduces rejection risk	Risk of tumor formation if undifferentiated cells remain; complex and costly production process	Research on heart disease and neurodegenerative diseases (e.g., Parkinson’s)	Requires stringent screening to prevent undifferentiated cells that could lead to tumorigenesis
Mesenchymal Stem Cells (MSCs)	Anti-inflammatory; immunomodulatory; multipotent; can be sourced from autologous tissues	Limited differentiation capacity; potential for immune response if not autologous	Osteoarthritis, degenerative diseases, age-related tissue repair	Variable potency and therapeutic outcomes based on source and culture conditions
Exosomes	Lower immune risk; cell-free therapy; simpler storage and administration	Limited therapeutic cargo size; less understood than full-cell therapies	Skin rejuvenation, joint repair, neurodegenerative disease applications	Limited targeting and delivery precision; requires rigorous validation for efficacy and safety
CRISPR Gene Editing	High precision in gene targeting; potential to edit genes associated with aging	Risk of off-target effects and unintended genetic changes; ethical concerns around germline editing	Research on anti-aging applications, disease prevention by modifying senescence-related genes	Delivery to specific cells is complex; regulatory and ethical challenges due to heritable genetic modifications

## Data Availability

Not applicable.
